# Role of the Critical Care Resuscitation Unit in a Comprehensive Stroke Center: Operations for Mechanical Thrombectomy During the Pandemic

**DOI:** 10.5811/westjem.18335

**Published:** 2024-06-20

**Authors:** Quincy K. Tran, Robinson Okolo, William Gum, Manal Faisal, Vainavi Gambhir, Aditi Singh, Zoe Gasparotti, Chad Schrier, Gaurav Jindal, William Teeter, Jessica Downing, Daniel J. Haase

**Affiliations:** * University of Maryland School of Medicine, Department of Emergency Medicine, Baltimore, Maryland; † University of Maryland School of Medicine, R Adams Cowley Shock Trauma Center, Program in Trauma, Baltimore, Maryland; ‡ University of Maryland School of Medicine, Department of Emergency Medicine, Research Associate Program in Emergency & Critical Care, Baltimore, Maryland; § University of Maryland Medical Center, Critical Care Resuscitation Unit, Baltimore, Maryland; ∥ University of Maryland, School of Medicine, Baltimore, Maryland; ¶ University of Maryland Medical Center, Department of Neurology, Baltimore, Maryland; # University of Maryland School of Medicine, Department of Neuroradiology, Baltimore, Maryland

## Abstract

**Introduction:**

Standard of care for patients with acute ischemic stroke from large vessel occlusion (AIS-LVO) includes prompt evaluation for urgent mechanical thrombectomy (MT) at a comprehensive stroke center (CSC). During the start of the coronavirus 2019 pandemic (COVID-19), there were reports about disruption to emergency department (ED) operations and delays in management of patients with AIS-LVO. In this study we investigate the outcome and operations for patients who were transferred from different EDs to an academic CSC’s critical care resuscitation unit (CCRU), which specializes in expeditious transfer of time-sensitive disease.

**Methods:**

This was a pre-post retrospective study using prospectively collected clinical data from our CSC’s stroke registry. Adult patients who were transferred from any ED to the CCRU and underwent MT were eligible. We compared time intervals in the pre-pandemic (PP) period between January 2018– February 2020, such as ED in-out and CCRU arrival-angiography, to those during the pandemic (DP) between March 2020–May 31, 2021. We used classification and regression tree (CART) analysis to identify which time intervals, besides clinical factors, were associated with good neurological outcome (90-day modified Rankin scale 0–2).

**Results:**

We analyzed 203 patients: 135 (66.5%) in the PP group and 68 (33.5%) in the DP group. Time from ED triage to computed tomography (difference 7 minutes, 95% confidence interval [CI] −12 to −1, *P* < 0.01) for the DP group was statistically longer, but ED in-out was similar for both groups. Time from CCRU arrival to angiography (difference 9 minutes, 95% CI 4–13, *P* < 0.01) for the DP group was shorter. Forty-nine percent of the DP group achieved mRS ≤ 2 vs 32% for the PP group (difference −17%, 95% CI −0.32 to −0.03, *P* < 0.01). The CART identified initial National Institutes of Health Stroke Scale, age, ED in-and-out time, and CCRU arrival-to-angiography time as important predictors of good outcome.

**Conclusion:**

Overall, the care process in EDs and at this single CSC for patients requiring MT were not heavily affected by the pandemic, as certain time metrics during the pandemic were statistically shorter than pre-pandemic intervals. Time intervals such as ED in-and-out and CCRU arrival-to-angiography were important factors in achieving good neurologic outcomes. Further study is necessary to confirm our observation and improve operational efficiency in the future.

Population Health Research Capsule What do we already know about this issue?

*During the pandemic (DP), the processes of care for patients in EDs were significantly delayed, compared to the pre-pandemic (PP) time.*

What was the research question?

*We sought to determine whether the process of care for patients with acute ischemic stroke from large vessel occlusion in the ED and the critical care resuscitation unit (CCRU) was affected during the pandemic.*

What was the major finding of the study?

*Total time in ED was similar at 157 minutes both PP and DP (p = 0.74), while DP time in the CCRU was 9 minutes shorter than PP.*

How does this improve population health?

*In-out ED time was one of the top predictors for outcome. Clinicians should expedite transfer of patients to thrombectomy.*

## INTRODUCTION

Prior research has shown that patients who sustain acute ischemic stroke from large vessel occlusion (AIS-LVO) face high rates of mortality and morbidity^1^ if they do not receive timely reperfusion therapy. Multiple studies have demonstrated that mechanical thrombectomy (MT) can improve neurologic outcomes for patients with AIS-LVO,^2^^–^^4^ and since 2015 MT has become the standard of care. Throughout the US, however, the technology and expertise required to perform MT are only available at approximately 216 comprehensive stroke centers (CSC),^5^ which also manage these critically ill patients in a specialized neurocritical care unit (NCCU). Therefore, patients with AIS-LVO who initially present to a hospital without MT capability require transfer to a CSC. Given the widely accepted association of time to reperfusion with neurologic outcomes (the adage “time is brain” very much applies), it is essential that both interhospital transfer and transfer to the interventional suite following arrival at the CSC are expeditious.^6^

The University of Maryland Medical Center (UMMC) in Baltimore, MD, is a CSC offering MT to patients with AIS-LVO throughout the state. To increase access to MT and avoid unnecessary delay of transfer due to bed unavailability at the NCCU, patients with AIS-LVO are transferred directly to the UMMC Critical Care Resuscitation Unit (CCRU), a six-bed resuscitation unit created to expedite transfer of patients with critical illness or time-sensitive diseases such as AIS-LVO.^7^^,^^8^ We have previously demonstrated that the CCRU is able to directly admit a majority of patients with AIS-LVO for MT when the NCCU at UMMC does not have available beds, while providing initial resuscitation and outcomes similar to patients who were transferred directly to the NCCU. Prior to the coronavirus 2019 (COVID-19) pandemic, up to 68% of patients transferred to UMMC for AIS-LVO were admitted first to the CCRU, while 32% were admitted directly to the NCCU.^9^

The onset of the COVID-19 pandemic affected the US healthcare system in many ways. During the early phase of the pandemic, staff shortages, personal protective equipment (PPE) requirements, and the lack of COVID-19 testing resulted in delays in the process of care for patients. Patients’ length of stay in the emergency department (ED) was longer than in the pre-pandemic period.^10^^,^^11^According to a Korean study, the essential time interval from ED triage to neuroimaging studies for patients with ischemic stroke was delayed when compared to the pre-pandemic period.^10^ This delay in the ED process of care is likely to have affected the outcome of patients transferred to CSCs for MT. It is not known whether the process of care for these patients with AIS-LVO transferred through the CCRU, which is specialized to expedite the transfer and treatment of patients with time-sensitive diseases, was also delayed during the pandemic.

In this pre-post pandemic study, we sought to compare the process of care for patients with AIS-LVO for both the ED and the CSCs, from ED triage to the CCRU, and subsequently to the MT suite. Acknowledging that the time interval from patients’ last-known-well period to the time of reperfusion (recanalization) is essential,^12^ we also investigated which time intervals following arrival to the ED were most important in determining patients’ neurological outcomes.

## METHODS

### Patient Selection

This was a retrospective study among adult patients transferred from any ED to the CCRU between January 1, 2018–May 31, 2021 for MT. Data for these patients with AIS-LVO was collected prospectively for our institutional stroke registry. We compared patients transferred between January 1, 2018–February 29, 2020 (pre-pandemic) with those who were transferred between March 1, 2020–May 31, 2021 (during the pandemic). The study was exempted from formal consent by the UMMC Institutional Review Board.

### Study Settings

The CCRU is a six-bed, intensive care unit (ICU)-based resuscitation unit that was created in July 2013 to expedite the transfer of patients with time-sensitive conditions^13^ to UMMC, a quaternary academic medical center offering a variety of time-sensitive interventions for critical patients, including MT, emergency cardiac and aortic surgery, extracorporeal membrane oxygenation, and neurosurgery. The CCRU has facilitated the transfer of over 1,500 patients per year, or up to 20% of total transfers, to our institution.^8^ Prior research has demonstrated that transfer through the CCRU was associated with more rapid transfer, defined as shorter intervals from transfer request to arrival at UMMC, than direct transfer to traditional inpatient critical care units.

The unit is staffed at all times by an onsite attending physician who is board certified in both emergency medicine (EM) and critical care medicine and an advanced practice practitioner (APP) with postgraduate training or experience in critical care. Fellow and resident physicians often rotate through the CCRU and work under the direct supervision of the CCRU attending. The nursing staff is composed of one charge nurse and four bedside nurses with at least two years of ICU experience; the charge nurse often participates in patients’ initial resuscitation and clinical care in addition to serving an administrative role. During the pandemic, there was no change in the basic staffing model of the CCRU.

Since the opening of the CCRU, patients with AIS-LVO who are considered candidates for MT by the Stroke Neurology team at UMMC are transferred to the NCCU or the CCRU (if there is no NCCU bed available, staffed, and ready at the time of transfer). Any regional emergency physician who has diagnosed a patient with an AIS-LVO and does not have in-house MT capabilities can directly connect to a multidisciplinary team responsible for determining eligibility for MT and coordinating appropriate care before, during, and after the procedure through the Maryland Access Center (MAC), which handles all transfers from other hospitals to the UMMC. This team includes the on-call attending physicians for the stroke neurology team, the NCCU, neuroradiology, and the CCRU.

During this discussion, eligibility for MT is determined, recommendations for initial care prior to thrombectomy (both at the sending facility and upon arrival at UMMC) are discussed, and—for eligible patients—arrangements for urgent thrombectomy and post-thrombectomy care (including “activation” of on-call but offsite teams during off hours) are initiated. For eligible patients, arrangements are made for prompt bed assignment in either the NCCU or the CCRU, depending on NCCU bed availability, and transport is arranged in conjunction with the referring facility, often coordinated by the MAC.

On CCRU arrival, patients are assessed immediately by the CCRU and stroke neurology teams. The CCRU team assesses hemodynamic stability and the need for airway protection, establishes adequate intravascular (and at times arterial) access, and initiates treatment of hypertension for patients who received thrombolytics prior to transfer. The stroke neurology team performs an initial National Institutes of Health Stroke Scale (NIHSS) assessment and confirms eligibility for MT. If eligibility is confirmed the patient, once stabilized, is transferred to the neuroradiology angiography suite for MT. Following thrombectomy, the patient is transferred either to the NCCU or the CCRU for further intensive stroke care. The patient is ultimately transferred to the NCCU when an appropriate bed is available.

This process, as well as the staffing and protocols of each involved medical team, had been maintained since before the pandemic and continued throughout the COVID-19 pandemic. During the pandemic, all patients transferred to the CCRU with thrombotic disease (such as ischemic stroke) were treated as a patient under investigation (PUI) for COVID-19 and remained so until results of a polymerase chain reaction (PCR) test became available. However, patients were still taken to the angiography suite immediately as indicated. When caring for any PUI, clinicians were required to use full PPE, including gowns, powered air-purifying respirators, and supplied-air hoods. Following a negative PCR, PPE requirements relaxed to require only gowns and N95 masks.

### Outcome

The primary outcome was the time interval between CCRU arrival and transfer to the angiography suite. This was selected a priori as a modifiable risk factor that reflects the process and efficiency of care within the CCRU. Our secondary outcome was the percentage of patients who achieved good neurologic recovery, defined as 90-day modified Rankin scale (mRS) score ≤2. The 90-day mRS score was collected prospectively by our stroke neurology team as part of required clinical stroke care for a CSC. For our intention-to-treat analysis, we categorized any patients who were lost to follow-up, such as patients in skilled nursing facilities, as mRS >3.

### Data Collection

Patient demographic data (age, gender, past medical history) was extracted from our electronic health records. Clinical data during the initial ED stay at the sending facility, such as initial vital signs, ED triage time, and time from triage to computed tomography (CT), was extracted using the paper records accompanying patients as part of the transfer process. Prior to data extraction, junior investigators who were not blinded to the study hypothesis were trained to collect data in sets of 10 patients’ charts until inter-rater agreement reached 90%. Data disagreement was adjudicated by a senior investigator. Data was extracted and entered into a standardized Excel spreadsheet (Microsoft Corporation, Redmond, WA).

### Data Analysis

We used descriptive analysis to express patient data as mean (±SD), median (interquartile [IQR]), or percentage. Prior to analysis, we assessed and analyzed histograms of continuous data distribution patterns with the Student *t*-test or Mann-Whitney U test as appropriate. Categorical data was analyzed via the Pearson chi-square test.

We performed time series analyses to examine the correlation of certain time intervals with new or cumulative cases of COVID-19. Data for global cases of COVID-19 was obtained from the website Statista.com on September 1, 2022.^14^ We performed analyses of different median time intervals to assess trends of different time intervals during the pandemic. The trend with the smallest values of mean absolute percentage error, mean absolute deviation, and mean squared deviation among four different algorithms (linear, quadratic, exponential growth, S-curve) was considered as having the best fit for the time series. To further assess the impact of the pandemic on operations of each stage of the patient’s care (from ED arrival to the angiography suite), we created a dummy variable, “presenting during pandemic,” for patients presenting between March 1, 2020–May 31, 2021.

We used the classification and regression tree (CART) method to identify predictors associated with patients’ neurological outcomes. The variables for the CART (Appendix 1) were identified a priori as known clinically important factors for patient outcome, according to literature and clinical consensus. The CART is a supervised, machine-learning technique that uses repetitive partitioning to identify a series of dichotomous splits (eg, 90-day mRS ≤2 vs 90-mRS ≥3) until the algorithm achieves “purity” where no further split is possible. The CART generated a tree of decision from the interactions between all the independent variables that we defined a priori. The algorithm assigns the most influential independent variable a relative variable importance (RVI) of 100%. Other important variables are assigned subsequent RVIs as percentages of the most important factor.

We assessed the discriminatory capability of the CART model using the area under the receiver operating curve (AUROC) analysis. An AUROC of 1.0 would have perfect discriminatory capability of predicting the dichotomous outcome. Our CART algorithm was performed with 10-fold cross-validation, a minimum of three counts per terminal node, and a maximum depth of 30 layers and 30 terminal nodes. The optimal tree was selected according to a balance between number of nodes and lowest miscalculation cost.

Additionally, we performed sensitivity analysis to assess whether the time intervals were important factors when analyzed with different groups of variables. In this sensitivity analysis, instead of using separate segments of time intervals, such as CCRU-to-angiogram suite, angiogram suite-to-groin puncture, and groin puncture -to-recanalization, we divided the overall time interval into ED in-and-out (covering the time from ED triage to transfer) and CCRU arrival-to-recanalization (Appendix 2).

We performed all descriptive analyses, time series and CART analyses via Minitab version 20 (Minitab LLC, State College, PA). All *P*-values < 0.05 were considered statistically significant.

## RESULTS

**Figure 1.**
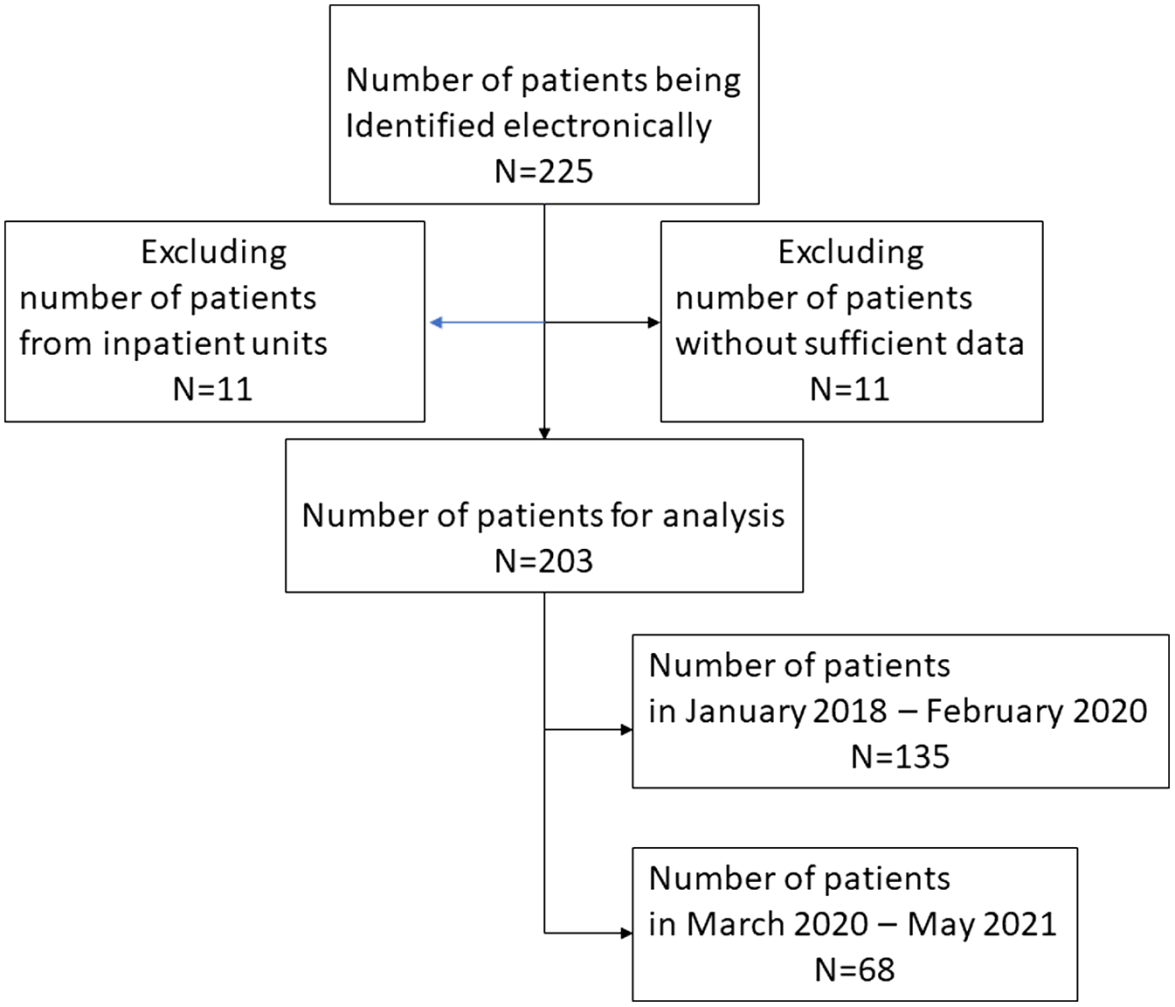
Patient selection diagram.

The study identified 225 patients during the study period; 22 patients did not meet inclusion criteria, and 203 were included in the final analysis (Figure 1). One hundred thirty-five (66.5%) patients with AIS-LVO were transferred from an ED to the CCRU between January 2018–February 2020 pre-pandemic, while 68 (33.5%) were transferred between March 2020–May 2021 during the pandemic. The mean age was 67 (±15) years (Table 1). Patients’ median NIHSS at CCRU arrival in the pre-pandemic period was similar to that of patients during the pandemic period (Table 1). Patients during the pandemic period had a higher percentage of occlusion from middle cerebral artery (59/68, 87%), compared to patients in the pre-pandemic period (97/143, 72%, difference 15%, 95% CI −0.26 to −0.04). A higher percentage of patients in the pandemic period achieved good 90-day neurological recovery (33/68, 49%) compared to patients in the pre-pandemic group (41/143, 32%, difference 17%, 95% CI −0.32 to −0.03).

**Table 1.** Patients’ demographics.

Variables	All patients	Pre-pandemic (1/2018–2/2020)	Pandemic (3/2020–5/2021)	Difference between groups		*P-*value
	N = 203	N = 135	N = 68	N	95% CI	
Age, mean (SD)	67 (15.15)	66 (14.94)	68 (15.57)	−1.89	(−6.42, 2.63)	0.41
Gender						
Female, N (%)	111 (55)	72 (53)	39 (57)	−0.04	(−0.18, 0.10)	0.66
Male, N (%)	92 (45)	63 (47)	29 (43)	0.04	(−0.10, 0.18)	0.66
IV thrombolysis, N (%)	89 (44)	63 (47)	26 (38)	0.08	(−0.06, 0.23)	0.3
NIHSS in ED, median [IQR]	17 [12–21]	17 [12–21]	16 [10–21]	1	(−1, 3)	0.35
NIHSS on CCRU arrival, median [IQR]	17.5 [12–21.25]	18 [14–21]	16 [11–23]	0	(−2, 2)	0.71
Occluded vessels, N (%)						
Internal carotid artery only	19 (9)	16 (12)	3 (4)	0.07	(0, 0.15)	0.12
Middle cerebral artery only	156 (77)	97 (72)	59 (87)	−0.15	(−0.26, −0.04)	0.02
Multiple vessels	28 (14)	22 (16)	6 (9)	0.07	(−0.02, 0.17)	0.2
Laboratory values, mean (SD)						
Sodium (mEq/L)	138 (3.29)	138 (3.16)	137 (3.36)	1.35	(0.38, 2.32)	0.007
Creatinine (mg/dL)	0.96 (0.81)	0.91 (0.34)	1.04 (1.32)	−0.13	(−0.46, 0.19)	0.41
International normalized ratio	1.14 (0.25)	1.15 (0.25)	1.11 (0.25)	0.03	(−0.04, 0.11)	0.37
Outcomes						
TICI 2c/3, N (%)	132 (65)	85 (63)	47 (69)	−0.06	(−0.2, 0.08)	0.44
90-day mRS 0–2, N (%)	74 (38)	41 (32)	33 (49)	−0.17	(−0.32, −0.03)	0.02
Mortality, N (%)	46 (24)	30 (23)	16 (24)	0	(−0.13, 0.12)	0.99

*CI,* confidence interval; *CCRU*, critical care resuscitation unit; *ED*, emergency department; *IV*, intravenous; *mEq/L*, milliequivalent per liter; *mg/dL*, milligrams per deciliter; *mRS*, modified Rankin scale; *NIHSS*, National Institute of Health Stroke Scale; *TICI*, thrombolysis in cerebral infarction; *TICI 2c*: near complete perfusion except for slow flow; *TICI 3*: complete antegrade reperfusion of the previously occluded target artery.

### Time Intervals

Overall, median interval (minutes) from last known well time to recanalization was similar for both groups (462 [326–986] vs 557 [371–984], difference 40, 95% CI −119 to 32), although last known well time to CCRU arrival (327 [221–682] vs 472 [279–869], difference 80, 95% CI 20–157, *P* = 0.001) and groin puncture (370 [270–752] vs 512 [332–911], difference 80, 95% CI 20–154, *P* = 0.01) were significantly longer in the pandemic group.

Patients in the pandemic group had a statistically longer time from ED triage to CT (difference 7 minutes, 95% CI −12 to −1) (Table 2). However, ED in-and-out times were similar in both groups (Table 2). During the pandemic, patients had statistically shorter time (minutes) between arrival at the CCRU and leaving the CCRU for the angiography suite (difference 9, 95% CI 4–13). Similarly, median interval (in minutes) from groin puncture to recanalization was statistically shorter during the pandemic (difference 9, 95% CI 2–17).

**Table 2.** Comparison of various time intervals for patients with cerebrovascular accident due to large vessel occlusion presenting for mechanical thrombectomy prior to or during the COVID-19 pandemic.

Variables	All patients	Pre-pandemic (1/2018–2/2020)	Pandemic (3/2020–5/2021)	Difference between groups		*P*-value
	N = 203	N = 135	N = 68	N	95% CI	
Intervals from LKW						
LKW to CCRU arrival	361 [243–724]	327 [221–682]	472 [279–869]	−80	(−157, −20)	0.001
LKW to groin puncture	403 [294–784]	370 [270–752]	512 [332–911]	−80	(−154, −20)	0.01
LKW to recanalization	483 [340–986]	462 [326–986]	557 [371–984]	−40	(−119, 32)	0.25
ED time intervals (minutes), median [IQR]						
Triage to CT scan results	25 [14–40]	21 [13–37]	30.5 [18.3–47]	−7	(−12,−1)	0.02
Triage to neurology consult at UMMC	65 [40–110]	68 [46–119]	57.5 [36–91.5]	11	(−1, 24)	0.09
Triage to IV thrombolysis (N = 91)	48 [31–72]	48 [29–70.5]	51 [33.5–74]	−1	(−13, 12)	0.79
Triage to leaving ED (ED in-out)	157 [125–211]	157 [119–221]	157 [131.3–202.8]	−3	(−20, 16)	0.74
Transfer request to CCRU arrival	111 [92–139]	106 [86–131]	121.5 [100–149]	−14	(−24, −3)	0.01
Time intervals after arrival at CCRU (minutes), median [IQR]						
CCRU arrival to thrombectomy suite	28 [18–40]	32 [21–44]	20.5 [14–33.8]	9	(4, 13)	0.01
Thrombectomy suite to groin puncture (minutes), median [IQR]	14 [11–19]	13 [10–17]	18.5 [13.25–22.75]	−5	(−7, −3)	0.01
Groin puncture to recanalization(minutes), median [IQR]	40 [23–70]	44 [27–73]	37 [19.25–55]	9	(2, 17)	0.01

*CCRU*, critical care resuscitation unit; *CT*, computer tomography; *ED*, emergency department; *IQR*, interquartile range; *IV*, intravenous; *LKW*, last known well; *UMMC*, University of Maryland Medical Center.

**Figure 2.**
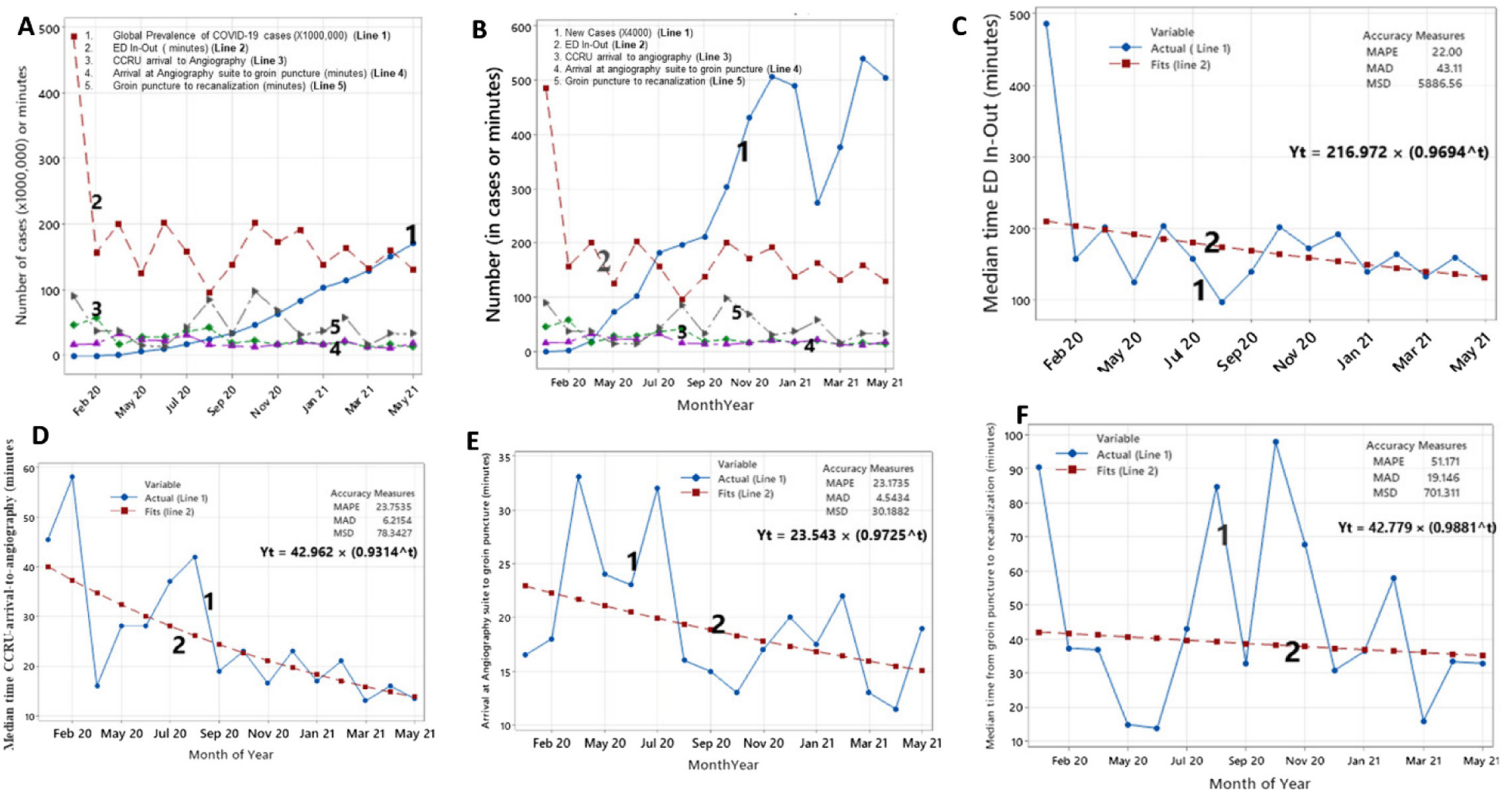
Time series analysis of different time intervals for patients with cerebrovascular accident due to large vessel occlusion (LVO) presenting for mechanical thrombectomy during the COVID-19 pandemic. Figure 2A. Time series analysis comparing different time intervals for treatment of cerebrovascular accident due to LVO and global prevalence of COVID-19 cases. Figure 2B. Time series analysis of prevalence of new COVID-19 cases and different time intervals for treatment of cerebrovascular accident due to LVO. Figure 2C. Trend analysis of time interval of ED in-and-out time for patients with cerebrovascular accident due to LVO over the course of the COVID-19 pandemic. Figure 2D. Trend analysis of time interval between CCRU arrival and arrival in the angiography suite for patients with cerebrovascular accident due to LVO presenting during the COVID-19 pandemic. Figure 2E. Trend analysis of time interval from arrival at the angiography suite to groin puncture for patients with cerebrovascular accident due to LVO occlusion presenting during the COVID-19 pandemic. Figure 2F. Trend analysis of time interval from groin puncture to recanalization for patients with cerebrovascular accident due to LVO presenting during the COVID-19 pandemic. *CCRU*, critical care resuscitation unit; *COVID-19*, coronavirus disease 2019; *ED*, emergency department; *MAPE*, mean absolute percentage error; *MAD*, mean absolute deviation; *MSD*, mean squared deviation; *IR*, interventional radiology.

We plotted median values of different time intervals with the number of total global cases of COVID-19 (Figure 2A) or total number of global new cases (Figure 2B). This time series suggested that the ED in-and-out time was most parallel with the number of new cases (Figure 2B, line 1 and line 2). Figures 2C–2F display different trend analyses for different time intervals between January 2020–May 2021. Overall, a downward trend of all time intervals toward May 2021 was observed.

### Classification and Regression Tree (CART) Analysis

**Figure 3.**
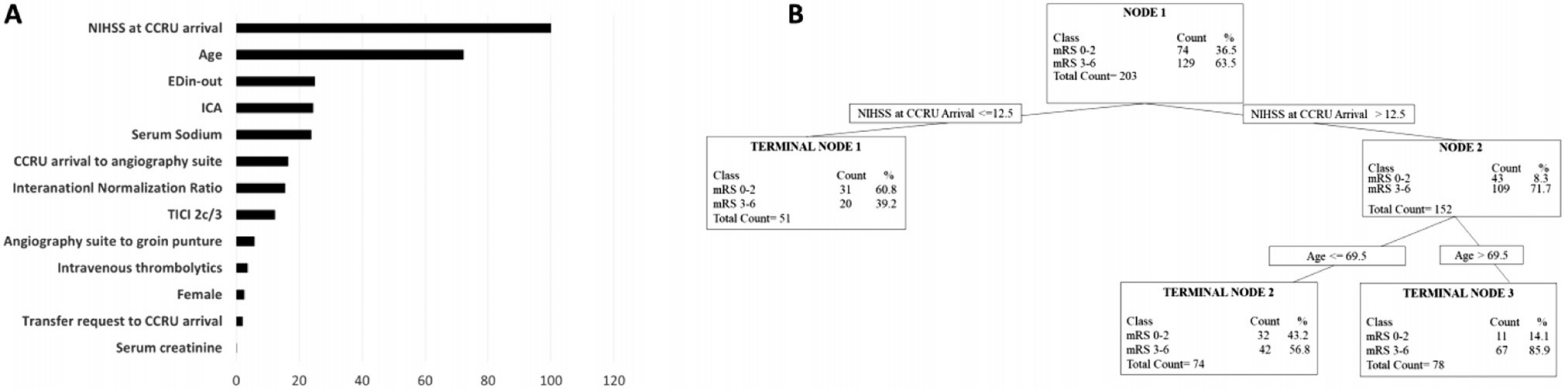
Relative variable importance (RVI) values and the tree diagram from the classification and regression tree (CART) analysis. Figure 3A. RVI from the CART analysis. The CART was used to assess important clinical factors and patients’ neurological outcome, defined as 90-day modified Rankin scale (mRS) 0–2. Figure 3B. The tree diagram from the CART analysis. The CART was used to assess important clinical factors and patients’ neurological outcome, defined as 90-day mRS 0–2.

The CART analysis identified that patients’ NIHSS at arrival at the CCRU was the most important predictor for poor neurological recovery at 90 days, as NIHSS was assigned a RVI of 100% (Figure 3A). The ED in-and-out time and CCRU arrival-to-angiography time were identified by the CART analysis as the third and sixth most important factors for good neurologic outcome, with reported RVI of 25% and 16.5%, respectively (Figure 3A). Patient’s NIHSS at CCRU arrival was responsible for the first split in the decision tree (Node 1, Figure 3B). If a patient’s age was greater than 69.5 years (Node 2), the patient was more likely to have poor neurologic recovery (Terminal node 3, Figure 3B). The only modifiable risk factors identified as “important” were median ED in-and-out and CCRU arrival-to-angiography times. The AUROC for the CART’s training dataset was good (0.72), as was the AUROC for the test dataset (0.58); misclassification cost was 0.63.

## DISCUSSION

Our findings suggest that despite previously noted impacts of the COVID-19 pandemic on multiple aspects of emergency and critical care, the care processes used to facilitate treatment with MT for patients with AIS-LVO were relatively unaffected, as were patient outcomes. Given the spoke-and-hub model of comprehensive stroke care frequently employed throughout the US, including at our center, treatment with MT requires rapid coordination of multiple teams and resources, often across multiple resources. We found that the only time interval during which patients experienced statistically significant delays was that from ED triage to CT scanner (although with a mean difference of only 7 minutes, it is unclear whether this delay conferred clinical significance). This suggests that once an LVO was identified, the care coordination systems previously developed to facilitate rapid transfer and treatment of these patients were able to operate efficiently despite the ongoing pandemic.

The philosophy that “time is brain” continues to be the prime consideration in the treatment of patients with AIS-LVO, and has led to a nationwide emphasis on efficiency, organization, and protocolization of stroke identification and treatment at each stage of care: in the community (via education initiatives promoting stroke recognition); among emergency medical services (EMS) professionals; in the ED; and in in-hospital settings across the country. The importance of these systems and organized care have been emphasized in clinical studies and national guidelines.^15^^,^^16^ The findings presented in this study support this emphasis as well: our CART analysis identified the time interval between CCRU arrival and arrival in the angiography suite and that between ED triage and departure for transfer as the most important modifiable risk factors in patients’ neurologic outcomes.

Although our finding is consistent with current consensus,^6^ it was in contrast to a previous study about time interval metrics in the ED.^5^ Scheving et al^5^ suggested that time intervals in the ED were not associated with patients’ 90-day outcome. However, the study by Scheving et al was restricted by a smaller number of ED patients undergoing MT and retrospective calculation of mRS. Our institution uses a highly coordinated and protocolized approach to facilitate prompt identification, transfer, and treatment of patients presenting to surrounding primary stroke centers who are candidates for MT. The expeditious transfer of patients with time-sensitive illness is a primary mission of the CCRU,^13^ and our group has previously demonstrated that the CCRU model is associated with shorter transfer times for patients with AIS-LVO to our institution.^9^

Our findings not only support previous recommendations that protocolized and organized care systems should be prioritized given an association with improved outcomes but highlight that such systems can promote standardized and efficient care even in the setting of large-scale disruptions and disasters, such as the COVID-19 pandemic. Patients transferred to our facility during the pandemic did not experience worse outcomes than those presenting pre-pandemic and—apart from time from ED triage to CT imaging, as noted above—did not experience significant delays in their care following ED arrival. Our time-series analysis found that, except for an initial slowdown in ED in-and-out time at the very beginning of the pandemic, which we believe is consistent with healthcare access issues experienced by patients during this early period and the outsized operational impact of the outbreak,^10^^,^^11^ each step of care for patients with AIS-LVO proceeded at a relatively constant (to slightly improving) rate following ED arrival, regardless of prevalence of total or new COVID-19 cases. While these trends were likely, at least in part, due to the relatively small number of AIS-LVO patients presenting to EDs during the early COVID-19 period, we believe they also reflect the resilience of stroke care protocols across multiple care settings.

Within certain areas of the hospital, the COVID-19 pandemic prompted the introduction of new care and coordination processes to meet the demands of an increasing volume of critically ill patients and ensure the safety of care team members when caring for patients with a highly communicable disease. These processes may have improved care coordination for patients without COVID-19 as well. For example, during the height of the COVID-19 pandemic, all transport clinicians were required to notify the CCRU team of their estimated time of arrival, to give team members time to don their PPE in preparation to receive the patients. For patients transferred for AIS-LVO, the stroke neurology and neuro-interventional teams received the same advanced notice, which allowed them to be present at the bedside when the patient arrived. After a quick assessment, eligible patients were then quickly moved from the CCRU to the angiography suite by the neuro-interventional team. Our study demonstrated relative reductions in the median times from CCRU arrival to angiography suite, and from CCRU arrival to recanalization overall, which may in part reflect the impact of these new protocols.

While we found that stroke processes of care in the ED and within the hospital were relatively unaffected by the pandemic, we did observe a significant increase in time from last known wellness to arrival at the CSC during COVID-19, highlighting the breakdown in the first step of the stroke “chain of survival”—activation of EMS. This is unsurprising given the emphasis on social distancing and resultant isolation during the pandemic. Although this risk factor is modifiable through improved public education and outreach, it is not a time interval that can be meaningfully impacted by hospital and ED processes, and thus was not included in our CART analysis. Multiple prior studies have demonstrated delays in presentation for stroke during the COVID-19 pandemic across the globe, thought to be related to delays in recognition of stroke symptoms or calling for help due to social isolation as well as fear of contracting COVID-19 in a healthcare setting.^17^^–^^20^ We anticipate that this breakdown may have had an even greater impact outside the scope of this study by reducing the percentage of AIS-LVO patients presenting within the “window” for MT. Because our study population included only patients transferred for thrombectomy, those patients would not be captured here.

## LIMITATIONS

Given the unique model of the CCRU as a well-resourced resuscitation unit dedicated to facilitating rapid transfer and critical care for patients with time-sensitive conditions, our results may not be generalizable. The pre-thrombectomy care provided in our CCRU population would be likely to occur in the ED at other facilities that do not have similar models, which may be more subject to the constraints imposed by COVID-19 (although our findings do not suggest this). However, our population was derived from more than 50 referring EDs within the regions; therefore, the time metrics from the ED to arrival to recanalization should still be applicable to other institutions. Since almost all our patients were transferred from other hospitals, a large percentage of the patients did not have Alberta Stroke Program Early CT (ASPECT) scores; therefore, we decided not to report the ASPECT score or use it in our analysis. The number of patients being transferred to the CCRU during the study period was relatively smaller than in the pre-pandemic period, which lowered the AUROC of our CART algorithm during the testing phase. Neither did we assess the COVID-19 vaccination status among patients and staff, which might have affected the CCRU staff’s preparedness when receiving patients.

## CONCLUSION

This study showed that the outcomes and initial care of patients with acute ischemic stroke from large vessel occlusion treated with mechanical thrombectomy were not affected by the COVID-19 pandemic at our comprehensive stroke center. This initial care spanned from ED arrival through identification of LVO, coordination of transfer to a CSC, and facilitation of rapid mechanical thrombectomy. Besides the patients’ intrinsic factors (NIHSS at arrival, age), the time intervals from ED arrival to transfer, and from CCRU arrival to arrival in the angiography suite, were identified as important, independent risk factors associated with 90-day modified Rankin scale. This highlights the importance of streamlined and protocolized care for patients with AIS-LVO eligible for mechanical thrombectomy and illustrates the role of a critical care resuscitation unit in promoting these care systems.

## Supplementary Information




