# Supplementary material for: Role of the Critical Care Resuscitation Unit in a Comprehensive Stroke Center: Operations for Mechanical Thrombectomy During the Pandemic
Source: West J Emerg Med. 2024 Jun 20;25(4):548–56. doi: 10.5811/westjem.18335 (PMC11254161; doi:10.5811/westjem.18335)
Supplement: Supplementary file 1 [file wjem-25-548-s001.docx]

**Appendix 2**. Sensitivity analysis using Classification And Regression Tree. In this sensitivity analysis, the total time intervals from patients’ CCRU arrival to recanalization was used, instead of individual segments. This sensitivity analysis confirmed that time intervals were still important factors for patients’ neurological outcome.


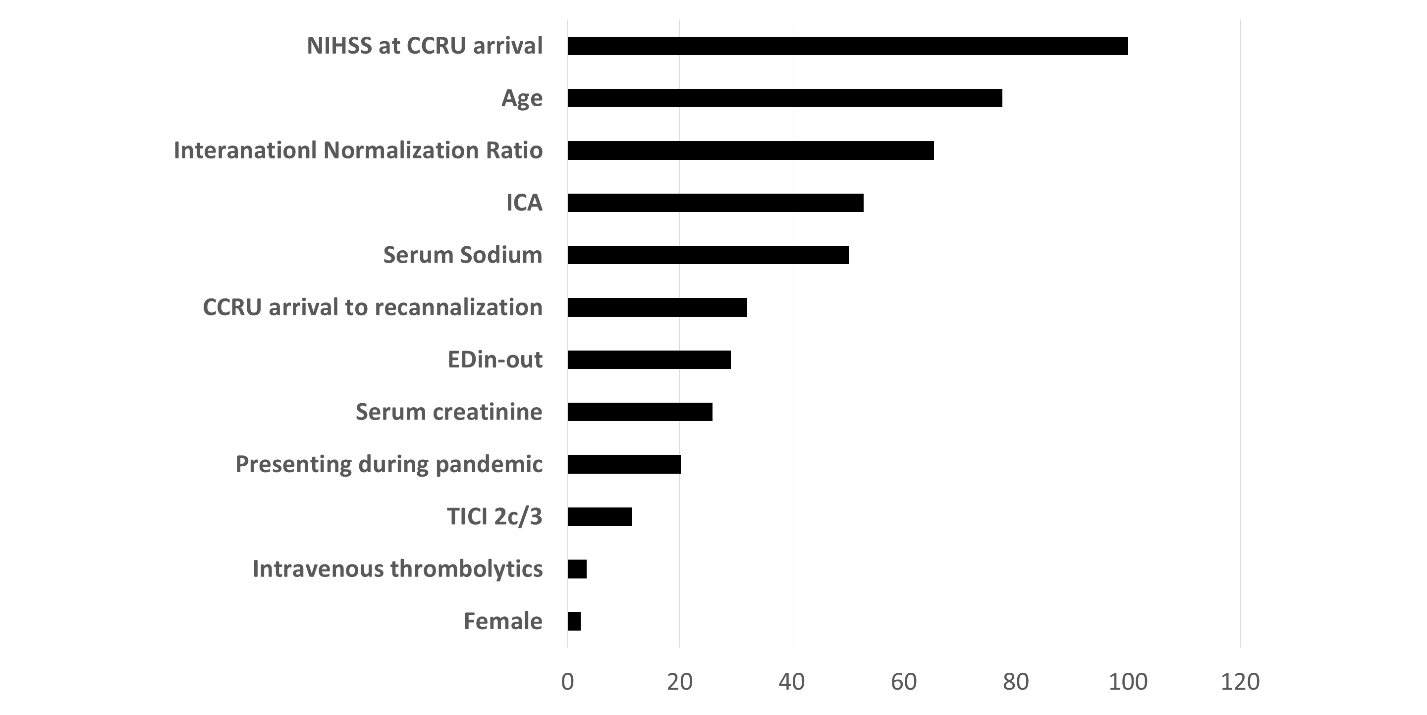


CCRU, Critical Care Resuscitation; INR, international normalized ratio; IR, angiography suite; NIHSS, National Institute of Health Stroke Scale; TICI, Thrombolysis in cerebral Infarction.
